# Novel high-efficiency 2D position-sensitive ZnS:Ag/^6^LiF scintillator detector for neutron diffraction

**DOI:** 10.1107/S1600576724002462

**Published:** 2024-05-10

**Authors:** Giacomo Mauri, G. Jeff Sykora, E. M. Schooneveld, S. C. Capelli, M. J. Gutmann, S. Howarth, S. E. Mann, F. Zuddas, N. J. Rhodes

**Affiliations:** a Science and Technology Facilities Council, ISIS Neutron and Muon Source, Rutherford Appleton Laboratory, Harwell, Oxford OX11 0QX, United Kingdom; Australian Nuclear Science and Technology Organisation, Lucas Heights, Australia

**Keywords:** neutron detectors, neutron diffraction, scintillator detectors, SXD diffractometer, high efficiency, wavelength-shifting fibre detector

## Abstract

Scintillator-based ZnS:Ag/^6^LiF neutron detectors have been under development at ISIS for more than three decades. Continuous research and development aim to improve detector capabilities, achieve better performance and meet the increasingly demanding requirements set by neutron instruments. As part of this program, a high-efficiency 2D position-sensitive scintillator detector with wavelength-shifting fibres has been developed for neutron-diffraction applications. The detector consists of a double scintillator-fibre layer to improve detection efficiency. Each layer is made up of two orthogonal fibre planes placed between two ZnS:Ag/^6^LiF scintillator screens. Thin reflective foils are attached to the front and back scintillators of each layer to minimize light cross-talk between layers. The detector has an active area of 192 × 192 mm with a square pixel size of 3 × 3 mm. As part of the development process of the double-layer detector, a single-layer detector was built, together with a prototype detector in which the two layers of the detector could be read out separately. Efficiency calculations and measurements of all three detectors are discussed. The novel double-layer detector has been installed and tested on the SXD diffractometer at ISIS. The detector performance is compared with the current scintillator detectors employed on SXD by studying reference crystal samples. More than a factor of 3 improvement in efficiency is achieved with the double-layer wavelength-shifting-fibre detector. Software routines for further optimizations in spatial resolution and uniformity of response have been implemented and tested for 2D detectors. The methods and results are discussed in this manuscript.

## Abstract 2

A novel high-efficiency 2D position-sensitive wavelength-shifting-fibre ZnS:Ag/^6^LiF scintillator detector has been developed at ISIS for single-crystal diffraction applications. More than a factor of 3 improvement in efficiency was achieved with this detector compared with clear-fibre scintillator detectors, and software routines for further optimizations in position sensitivity and uniformity have been implemented.

## 1. Introduction

Over the past three decades, ISIS has developed and deployed ZnS:Ag/^6^LiF scintillator-based neutron detectors for various neutron instruments (Rhodes *et al.*, 1997 ▸, 2004 ▸; Sykora *et al.*, 2015*a* ▸,*b* ▸; Mauri *et al.*, 2021 ▸) including reflectometers, single-crystal/powder diffractometers and inelastic neutron spectrometers. In general, neutron detectors for single-crystal diffraction require 2D position sensitivity, large-solid-angle coverage and high detection efficiency to maximize the accessible volume of reciprocal space. 2D position-sensitive detectors allow Bragg reflections in multiple crystallographic orientations to be measured simultaneously, hence increasing the data collection rate and enabling studies of smaller samples or samples with larger, more complex structures (Keen *et al.*, 2006 ▸; McIntyre, 2015 ▸). Moreover, in a time-of-flight (ToF) diffraction instrument, using the Laue technique, these volumes are completely resolved in three dimensions. Following Bragg’s law, the *d* spacing is directly proportional to the neutron wavelength or ToF, and hence all harmonics of a Bragg reflection can be recorded separately in a single measurement. The available neutron flux on the sample along with the solid-angle coverage and detection efficiency of the detector array are limiting factors in optimizing the instrument performance and the measurable sample volumes. In order to measure smaller samples or samples with complex structures, higher flux and/or higher detection efficiency is required. One of the first 2D scintillator-based neutron detectors for single-crystal diffraction was developed at ISIS (Rhodes *et al.*, 1997 ▸) for SXD (Keen *et al.*, 2006 ▸). Similar detectors have been developed at other neutron facilities over the past few years: large-area wavelength-shifting-fibre scintillator detectors (Crow *et al.*, 2004 ▸; Wang *et al.*, 2011 ▸) and neutron Anger cameras (Riedel *et al.*, 2015 ▸) at SNS, and several 2D neutron detectors using ^10^B/ZnS:Ag and 6Li/ZnS:Ag screens coupled to wavelength-shifting fibres at J-PARC (Hosoya *et al.*, 2009 ▸; Sakasai *et al.*, 2009 ▸; Nakamura *et al.*, 2012 ▸, 2017 ▸; Kawasaki *et al.*, 2014 ▸). Other neutron-detector technologies have been developed and employed for neutron diffraction: gaseous-based neutron detectors including the microstrip gas chamber and curved multiwire proportional chambers (Buffet *et al.*, 2005 ▸) at the ILL for the D20 and D1B diffractometers (Hansen *et al.*, 2008 ▸; Orench *et al.*, 2014 ▸), and boron-based detectors (Modzel *et al.*, 2014 ▸) for diffractometers at ESS (Stefanescu *et al.*, 2017 ▸, 2019 ▸). The current performance of SXD, the single-crystal ToF neutron diffractometer at ISIS, is limited by the available flux provided by the water moderator and the detector efficiency, which is approximately 15% at 1 Å. These limit the minimum measurable sample size to the order of 5 mm^3^ with unit-cell sizes of 5000–8000 Å^3^. The SXD detector array consists of 11 modules each with an active area of 192 × 192 mm and a square pixel size of 3 × 3 mm, providing a solid-angle coverage of about 50% of the scattered neutrons from the sample. Increasing the detector efficiency by a factor of 3 will allow smaller samples (<1 mm^3^) to be measured and structures of increasing complexity (unit-cell volume 10000–30000 Å^3^) to be characterized, extending the instrument capabilities to address new scientific areas. A novel double-layer wavelength-shifting-fibre (WLSF) ZnS:Ag/^6^LiF scintillator detector that achieves such performance is investigated in this paper. The design of this detector is presented, together with the efficiency measurements and calculations obtained with both a single-layer detector and a prototype detector in which the two layers of the detector could be read out separately. The double-layer module has been produced and installed on SXD. A spherical NaCl crystal was used to perform a direct comparison between the current and newly developed detectors. Further improvement in position resolution and uniformity can be implemented by employing a position-interpolation algorithm (Mauri *et al.*, 2021 ▸) and a uniformity-threshold correction. These methods have been tested with the detector and the results are discussed. SXD will undergo a detector upgrade which consists of replacing the 11 clear-fibre scintillator detectors (Rhodes *et al.*, 1997 ▸) with these novel double-layer WLSF ZnS:Ag/^6^LiF detectors. The development of such a detector with high efficiency, large area coverage, tunable position sensitivity and reasonable costs compared with other technologies paves the way to future instrument upgrades elsewhere at ISIS.

## 2. Double-layer detector design and characteristics

**Figure 1:**
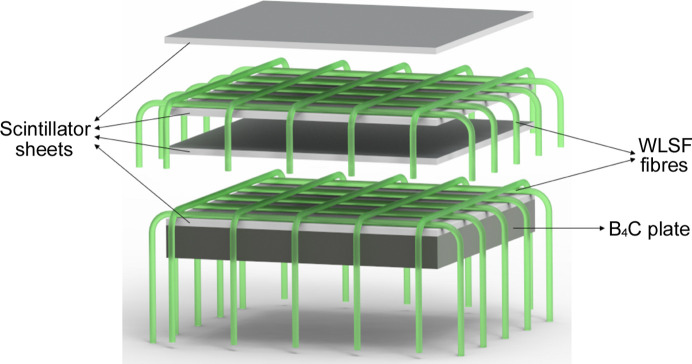
Sketch of the double-scintillator-fibre layer arrangement. A single layer consists of two orthogonal fibre planes (1 mm ø, 3 mm pitch) sandwiched between scintillator sheets (450 µm-thick) with reflective foils (50 µm-thick) and a 5 mm-thick B_4_C plate to reduce neutron background.

The novelty of the detector lies in the scintillator-fibre arrangement. As shown in Fig. 1 ▸, the detector is made up of two scintillator-fibre layers. Each layer consists of two orthogonal fibre planes placed between two 0.45 mm-thick, 2:1 ZnS:Ag to ^6^LiF ratio, scintillator sheets from Scintacor (https://scintacor.com/). The fibre array for both *X* and *Y* directions consists of 64 Y-11 S-type fibres with 300 p.p.m. dye concentration and a diameter of 1 mm from Kuraray (https://www.kuraray.com/). The fibre pitch is 3 mm. Thin reflective foils (50 µm thick) are attached to the front and back scintillator in each layer to eliminate light cross-talk between the two layers and improve light-collection efficiency. A 5 mm-thick B_4_C resin plate is placed at the back of the two layers to reduce neutron background from other sources. The air gap between the scintillator and fibres is minimized, with a nominal spacing of <100 µm, so that the effect of the air gap on light spread is negligible.

In this detector, a neutron is absorbed by a ^6^Li ion producing two charged particles: an α particle and a ^3^H. These particles ionize the ZnS:Ag which then emits broad-band blue light centred on 450 nm. Subsequently the light is absorbed by the wavelength-shifting fibres, re-emitted at longer wavelengths and transmitted to a Hamamatsu (https://www.hamamatsu.com/us/en.html) H14220A 64 channel multi-anode photomultiplier tube (MAPMT) with a green extended photocathode (Sykora *et al.*, 2015*a* ▸; Mauri *et al.*, 2021 ▸). Each plane of fibres (*X* and *Y*) is connected to its own MAPMT. A total of two 64-channel photomultiplier tubes (PMTs) are used to read out 4096 pixels, in contrast to the 32 single-anode PMTs employed in the current clear-fibre detector modules on SXD. A discriminator board with a field-programmable gate array (FPGA) for signal processing, coincidence identification and event positioning is located at the back of the detector. Note that the count-rate capability is limited by the timing properties of the scintillator and the subsequent signal processing to eliminate the possibility of counting a single neutron more than once. The light decay per neutron event in ZnS:Ag/^6^LiF ranges up to hundreds of microseconds. A local peak rate of 16 kcps and a global rate around 60 kcps are currently achievable for these detectors. A more detailed description of the signal processing and count-rate limitations can be found in previous work (Sykora *et al.*, 2012 ▸; Mauri *et al.*, 2021 ▸). A comparison of the detector characteristics between a clear-fibre single ZnS:Ag/^6^LiF sheet detector, a single-layer WLSF detector and a double-layer WLSF detector is provided in Table 1 ▸. The ^6^Li density of the scintillator, as quoted by Scintacor, is approximately 1.5 × 10^22^ atoms cm^−3^, which corresponds to a 72% absorption efficiency for the single-layer detector at 1.8 Å. Thus, the efficiency of 64% reported in Table 1 ▸ is approximately 90% of the calculated absorption. The new detector has a higher performance in count-rate capability, detection efficiency and position sensitivity at a fraction of the cost of a clear-fibre detector.

A direct comparison with the clear-fibre detector is reported in Section 3, highlighting the efficiency improvements, along with the future implementation to increase overall detector performance.

## 3. Results and discussion

### 3.1. Efficiency

**Figure 2:**
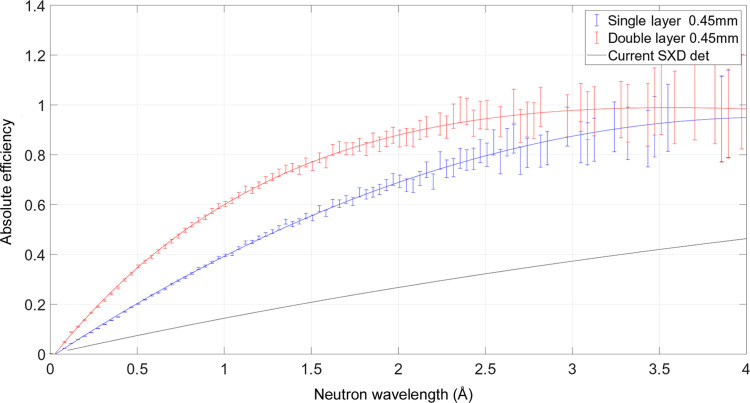
Efficiency of different scintillator configurations. The current SXD detector efficiency is the theoretical calculation of absorption efficiency for the single scintillator sheet (black curve). The measured efficiency of the single-layer (0.45 mm-thick scintillator front and back) and the double-layer detectors are shown as the blue and red curves respectively.

The detection efficiency is the most relevant performance improvement for the SXD upgrade project. The efficiency measurements were performed on the EMMA beamline at ISIS. This instrument is used to test beamline equipment including shielding materials, detectors, data-acquisition electronics and instrument software. EMMA is on a room-temperature water moderator with peak flux occurring at 1 Å (81.8 meV). A single-layer WLSF scintillator detector and a standard 1 inch-diameter ^3^He-tube filled to 6 bar pressure were measured in addition to a prototype detector with two scintillator-fibre layers. The prototype was built with independent read-out for the two layers so that the contribution to the efficiency of each layer could be investigated separately. The measurements were performed by placing the detectors in similar positions and using the same slit sizes to ensure the same incident neutron flux on all detectors. The detectors were shielded with B_4_C to reduce neutron scattering background. The theoretical efficiency for the current clear-fibre detector on SXD (ε) was calculated on the basis of the absorption efficiency of a 0.45 mm-thick ZnS:Ag/^6^LiF scintillator as a function of neutron wavelength (λ) as 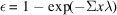 , where Σ is the macroscopic cross section and *x* is the thickness of the material. The result is depicted in Fig. 2 ▸ by the black curve. The absolute efficiency of the single-layer WLSF detector (ε_1_) was calculated with respect to the known efficiency of the 6 bar ^3^He tube ( 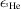 ) by comparing the spectra recorded with the measurement setup described above: 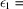 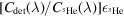 , where *C*_det_(λ) and 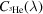 are the distributions measured, as a function of λ, for the single-layer WLSF detector and the ^3^He gas tube, respectively. The efficiency ε_1_ is shown in Fig. 2 ▸ as the blue curve. The absolute efficiency of the double-layer WLSF prototype (red curve in Fig. 2 ▸) was obtained following the same calculation procedure as for the single-layer detector. By comparing the red and black curves in Fig. 2 ▸, more than a factor of 3 improvement is expected at shorter wavelengths (<2 Å) and more than a factor of 2 up to 4 Å with the double-layer WLSF detector with respect to the current detector installed on SXD. An absolute efficiency of 85% for thermal neutrons at 2 Å is achieved with the double-layer WLSF detector, which is about 30% more than the efficiency of a single-layer WLSF scintillator detector.

**Figure 3:**
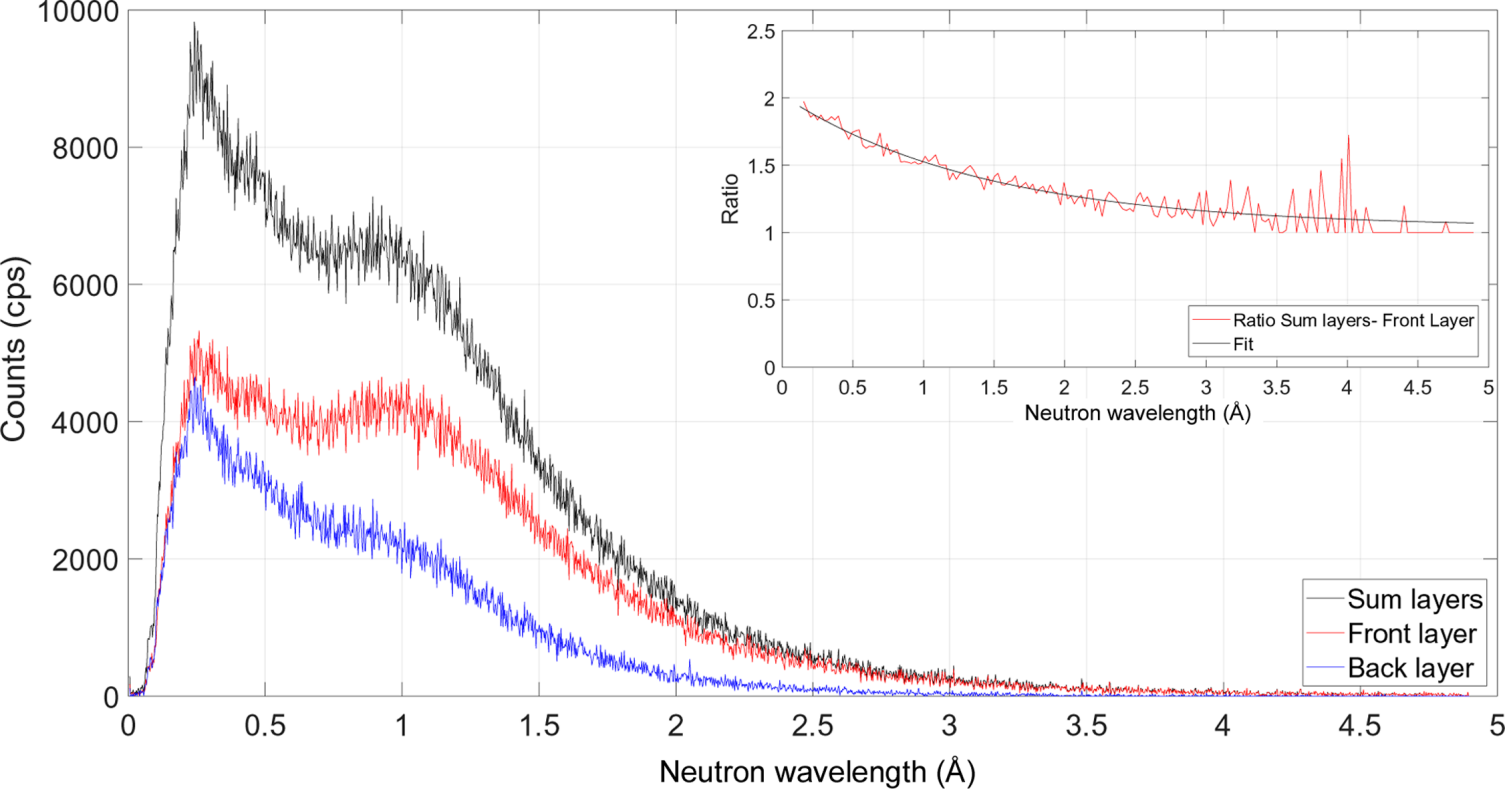
Incident spectrum with respect to neutron wavelength of the double-layer prototype. The contribution of the two layers summed together is represented by the black curve. The red and blue distributions correspond to the front and back layers, respectively. The ratio between the distributions of the layers summed together (black curve) and the front layer (red curve) is depicted in the inset plot together with the exponential fit.

**Figure 4:**
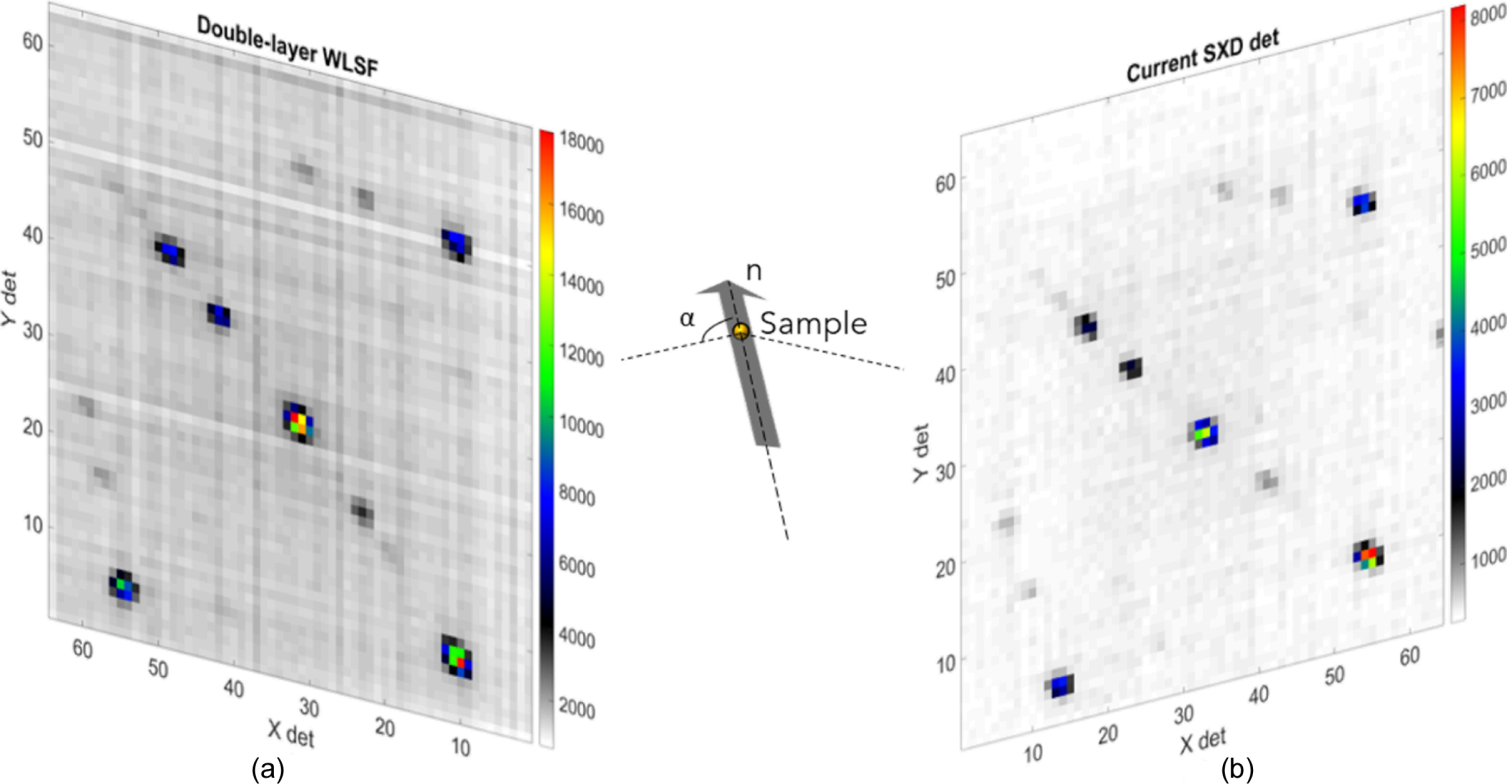
Laue diffraction image for an NaCl spherical crystal integrated in ToF with (*a*) the double-layer WLSF detector and (*b*) the current SXD detector. In order to obtain the same reflections on both detectors, the sample was measured at a fixed position and rotated 37.5° anti-clockwise for the current SXD detector measurements. The two plots are depicted in a sketch to illustrate the geometry and positions of the two detectors on the SXD instrument. The angle α is defined in equation (1). The colour bars represent the ToF integrated counts on a linear scale.

Fig. 3 ▸ shows the measured count-rate distributions of the prototype detector as a function of neutron wavelength for the two layers summed together (black curve) and the front and back layers separately as red and blue curves, respectively. The inset plot reports the ratio between the summed-layer and the front-layer distributions, together with the exponential fit 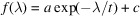 . The increase in efficiency between the single- and double-layer detectors, shown in Fig. 4 ▸, is in agreement with the value of the ratio reported in the inset plot in Fig. 3 ▸.

### 3.2. Peak count efficiency by time-resolved Laue diffraction

The time-resolved Laue diffraction technique exploits the wavelength discrimination of a pulsed source and 2D position-sensitive detectors to identify several Bragg reflections simultaneously in a single measurement. Using 2D position-sensitive detectors allows the information of an event to be recorded as a triplet (*X*, *Y*, ToF). The spatial coordinates (*X*, *Y*) identify the positions of events on the detector, while the ToF coordinate represents their time of arrival. The data can be represented by 2D plots as a function of (*X*, *Y*) and as a function of either the horizontal or the vertical spatial coordinate and ToF (*X*/*Y*, ToF). A spherical, 6 mm-diameter NaCl crystal with a face-centred cubic structure was measured to compare the intensities of reflections at similar wavelengths between the current clear-fibre detector and the double-layer WLSF detector. The two detectors were in the equatorial plane at longitude Θ = ± 142.5° with respect to the sample and at sample-to-detector distances *L*_2_ of 0.225 and 0.24 m, respectively. In order to get the same reflections on both detectors, the NaCl crystal was measured at two positions, one rotated 37.5° with respect to the other. The rotation angle is determined by the arrangement of the two detectors around the sample. Fig. 4 ▸ shows the Laue diffraction image integrated in ToF of the Bragg peaks recorded with the double-layer WLSF detector [Fig. 4 ▸(*a*)] and with the current SXD detector after performing the sample rotation [Fig. 4 ▸(*b*)]. The two plots are depicted in a sketch to illustrate the geometry and positions of the two detectors on the SXD instrument. The direction of the incident neutron beam and sample are shown in the sketch as well. The central reflection, located at (*X*, *Y*) coordinates (32, 28) for the double-layer WLSF detector and (33, 27) for the clear-fibre detector, is used to compare the efficiency of the new double-layer WLSF detector with the efficiency of the detector currently installed on SXD.

**Figure 5:**
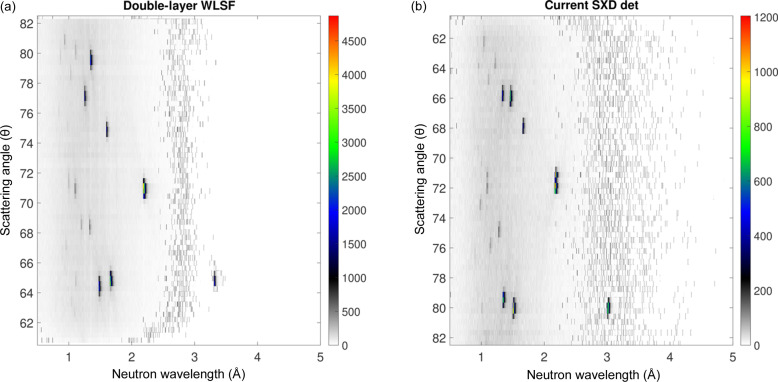
2D images of the Bragg reflections in (λ, θ) space for (*a*) the double-layer WLSF detector and (*b*) the current SXD detector. The colour bars represent counts on a linear scale.

**Figure 6:**
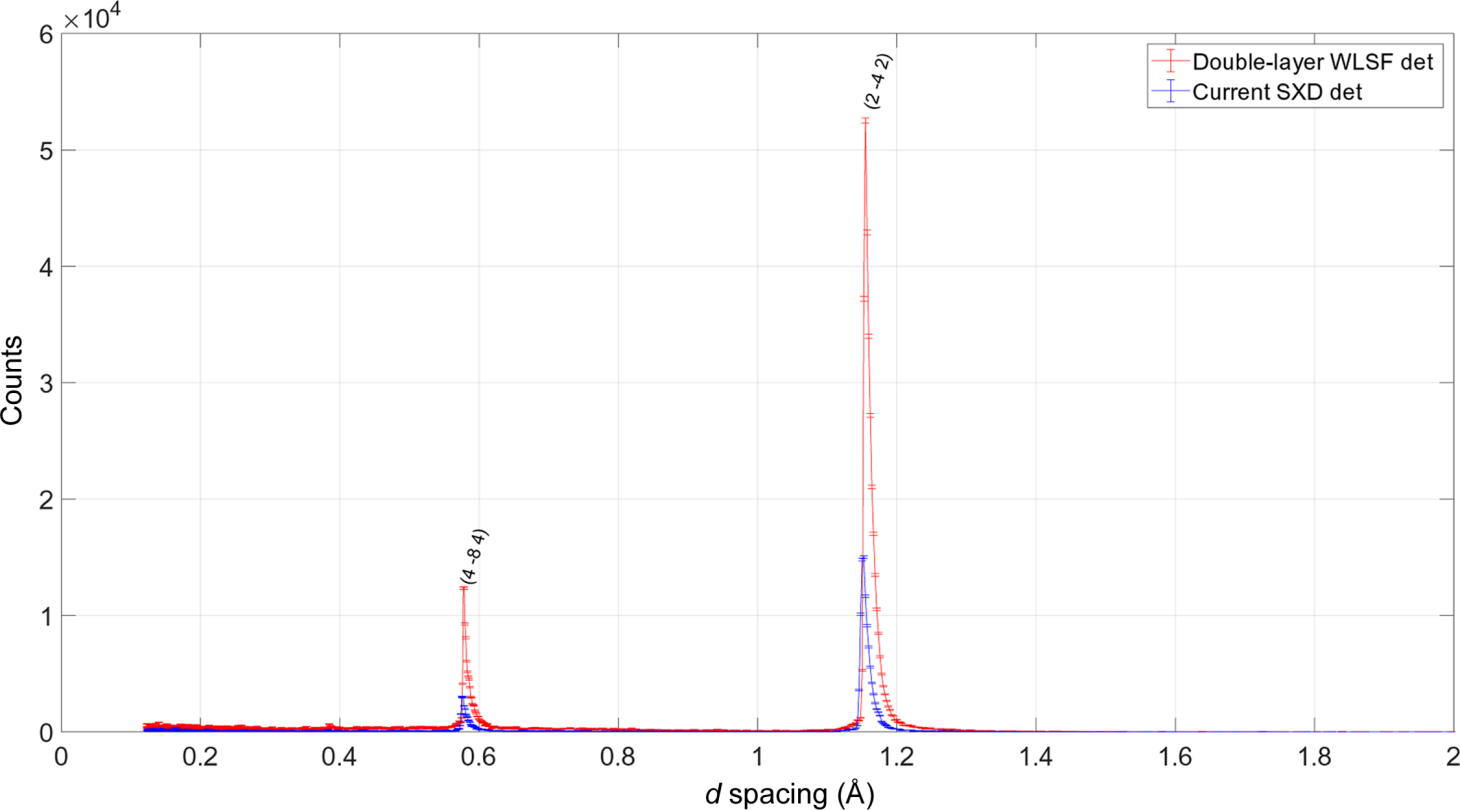
Intensities of 2 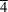 2 and 4 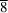 4 Bragg reflections integrated over a 3 ×  5 detector pixel area around the (32, 28) Bragg spot recorded on the double-layer WLSF detector (red curve) and the corresponding peak recorded with the current SXD detector centred at pixel (33, 27) (blue curve). The intensities are normalized by measurement time and solid-angle coverage.

The interplanar spacing (*d* spacing) between atoms is obtained by Bragg’s law, *d* = λ/[2sin(θ)], which defines the position of the reflections based on the neutron wavelength and scattering angle. The neutron wavelength is calculated from the ToF knowing the instrument flight path (*i.e.* the source-to-detector distance) while the spatial coordinates of the detector, *X* and *Y*, can be used to calculate the angles within and out of the equatorial plane, α and φ, respectively. The scattering angle θ is derived by combining these two angles as cos(2θ) = cos(α)cos(φ). The expressions for α and φ are reported in equations (1) and (2): 

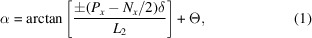




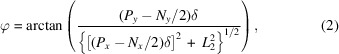

where *N_x_* = *N_y_* = 64 are the horizontal and vertical numbers of detector pixels, δ = 3 mm is the pixel size, *P_x_* and *P_y_* represent the positions of pixels 1 to *N_x_* and *N_y_*, and *L*_2_ and Θ identify the position of the centre of the detector on the instrument. The ± sign in equation (1) depends on the sign of *P_x_* with respect to Θ. The 2D image of the Bragg reflections in (λ, θ) space is depicted in Fig. 5 ▸(*a*) for the double-layer WLSF detector and Fig. 5 ▸(*b*) for the current SXD detector. The orders of the Bragg reflections are separated in λ and can be identified for each of the peaks recorded in Fig. 4 ▸. It is possible to distinguish the first and second orders of the reflection recorded at pixel (32, 28), corresponding to θ = 71.2°, at λ ≃ 2.2 and 1.1 Å, respectively. The intensity of the reflection is given in Fig. 6 ▸ as a function of *d* spacing for the double-layer WLSF detector in red and for the current SXD detector in blue. Note that the reflection is excited at a slightly different wavelength in the two detectors (<0.5% difference). This difference should not have a significant impact on the overall comparison. In order to further reduce this effect, the intensity was integrated over a 3 × 5 pixel rectangle around the detector pixel with highest intensity; moreover, it was normalized by measurement time and solid-angle coverage so that a direct comparison can be performed. The peak counting efficiency increases by a factor of 3.5 for the first-order reflection 2 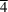 2 at *d* = 1.15 Å and it is up to a factor of 4 for the second-order peak 4 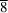 4 at *d* = 0.62 Å. The measured increase in peak counting is in agreement with the efficiencies reported in Section 3.1. The absolute efficiency of the newly developed double-layer WLSF detector is around 60% at 1 Å and up to 80% at 2 Å. The enhanced detector performance will enable faster measurements and increase the throughput of the single-crystal-diffraction science programme at ISIS.

### 3.3. Future performance implementations

Spatial resolution and uniformity are important features that can further improve the overall performance of the detector. A better spatial resolution allows us to spatially resolve Bragg reflections in very close proximity, extending the size of the crystal unit cell that can be investigated. A more uniform response will enhance the discrimination of low-intensity signals above the background. A position-interpolation algorithm and a variable-threshold correction method have been developed and implemented in general for 2D detectors. The results from both methods will be shown in the following sections.

#### 3.3.1. Improvement in position sensitivity using the centre of gravity

**Figure 7:**
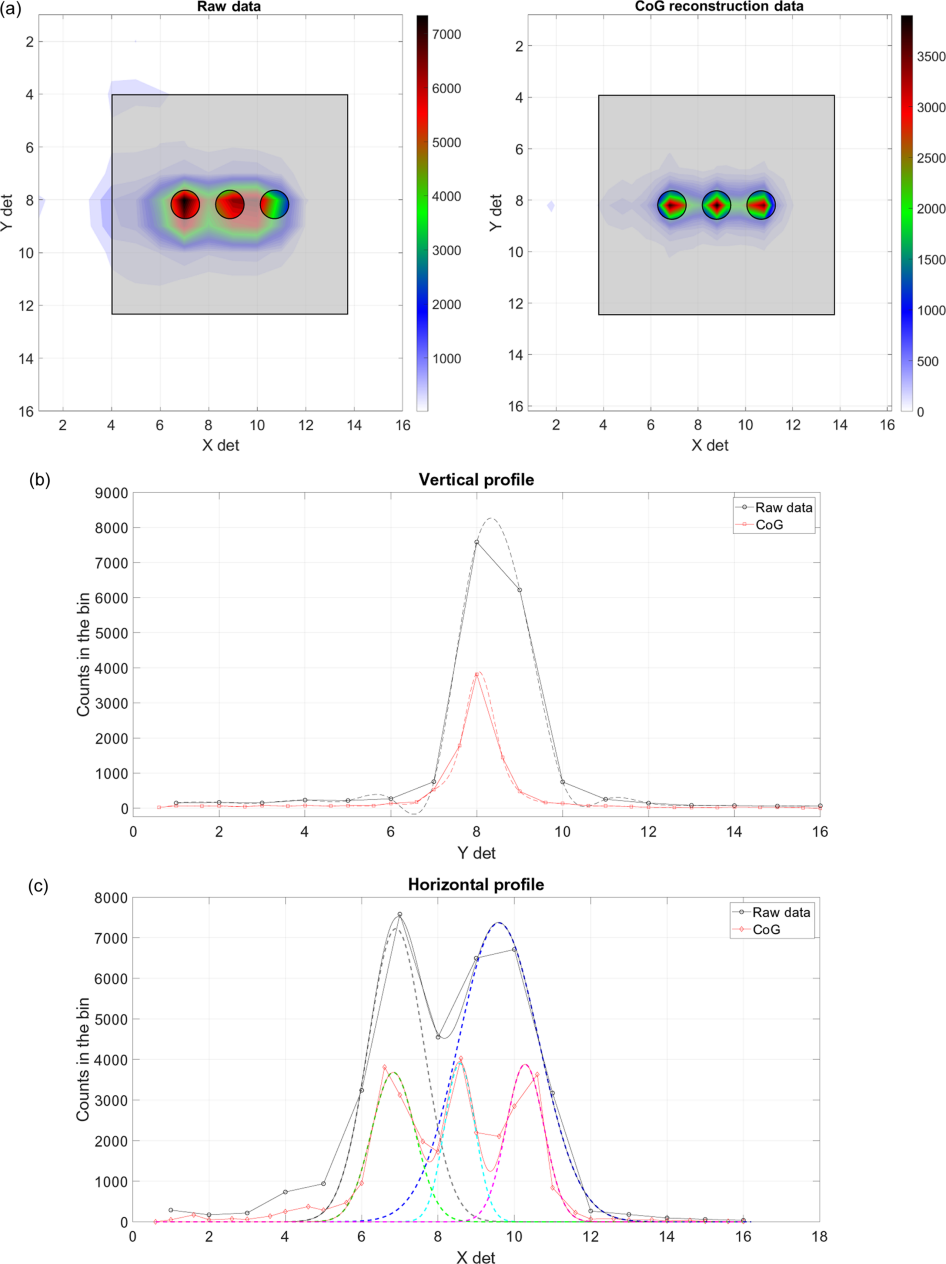
(*a*) 2D images of three 2 mm holes in a B_4_C mask for the raw data (left) and the data analysed using the CoG position-interpolation algorithm (right). A sketch of the mask shows the positions of the three holes and highlights the effectiveness of the CoG algorithm. The colour bars represent the counts integrated by time on a linear scale. (*b*) Vertical profile of the three-hole mask, for the raw data (black) and for the CoG processed data (red). (*c*) Horizontal profile of the three-hole mask for the raw data (black) and for the CoG processed data (red). The three peaks corresponding to the three holes are clearly distinguished when the CoG is applied, but only two peaks are visible in the raw data.

A centre-of-gravity (CoG) position-interpolation algorithm has been developed to improve the spatial resolution of WLSF scintillator detectors. A detailed description of the method can be found in previous work (Mauri *et al.*, 2021 ▸). The algorithm was first developed and tested with linear detectors. The implementation for 2D position-sensitive detectors is now complete. The investigation of the position-interpolation algorithm was performed with a smaller version of the 2D WLSF scintillator detector developed for the SXD detector upgrade. The detector was tested on the EMMA test beamline at ISIS. A B_4_C mask with three horizontal holes of 2 mm diameter on a 6 mm pitch was placed in front of the detector active area. Note that these measurements are per­formed to test the effectiveness of the position-interpolation algorithm for 2D detectors, not to define the minimum achievable position resolution that can be obtained. The 2D images of the raw data and the data processed with the CoG algorithm are shown in Fig. 7 ▸(*a*). A sketch of the mask is depicted on the 2D images to highlight the effectiveness of the CoG method. The three holes are clearly resolved when applying the position-interpolation algorithm, though it is not possible to distinguish them in the raw data image.

A section over the *X* dimension returns the profile of the three-hole mask in the vertical direction, see Fig. 7 ▸(*b*). Similarly, in Fig. 7 ▸(*c*), the horizontal profile of the mask is shown for a fixed value of *Y*. The vertical and horizontal profiles are shown in black for the raw data and in red for the data analysed with the CoG algorithm. A Gaussian fit is applied to the data to calculate the FWHM of each curve. The dotted lines in Figs. 7 ▸(*b*) and 7 ▸(*c*) represent the Gaussian fits. All curves are depicted as counts in the bin, since the number of bins used for the CoG method is double that used for the standard analysis. The FWHM values are reported in Table 2 ▸. More than a factor of 2 improvement is achieved by applying the CoG position-interpolation algorithm; moreover, only two out of the three holes are distinguishable with the raw-data processing.

The implementation of the CoG algorithm in the FPGA of the front-end electronics of the double-layer WLSF detector for on-board real-time calculation is ongoing. The firmware under development will allow data acquisition either with or without position interpolation based on the instrument requirements.

#### 3.3.2. Variable-threshold uniformity correction

**Figure 8:**
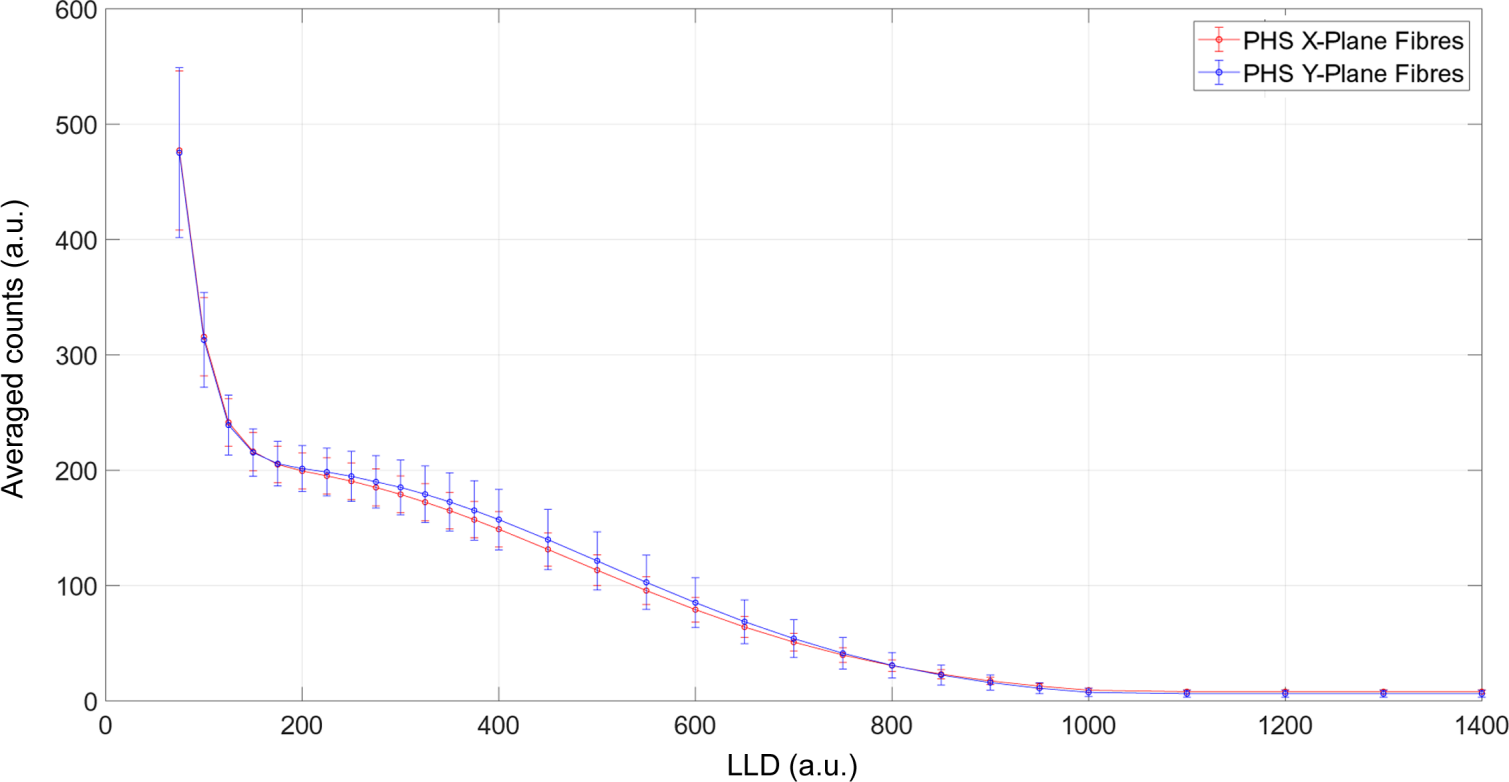
Average counts for the *X* and *Y* fibre planes of the double-layer WLSF ZnS:Ag/^6^LiF detector as a function of lower-level discrimination (LLD) are shown as red and blue curves, respectively. The error bar represents the calculated standard deviation per LLD as ±σ.

As described in Section 2, the detector has two orthogonal fibre planes, each of them consisting of 64 fibres that are connected to two 64-channel MAPMTs. This means that each fibre is connected to one PMT channel and a simple one-to-one fibre-to-PMT-channel coding is used. The detector uniformity is affected by several factors, mainly the wide dynamic range of the pulse heights recorded, quantum-efficiency variation of PMT channels and possible defects in the fibres leading to higher light loss along them. The single-photon pulse processing developed for these detectors (Sykora *et al.*, 2012 ▸, 2015*a* ▸; Mauri *et al.*, 2021 ▸) results in an effective photon-density profile, photons per unit time, which allows neutron and gamma events to be discriminated by applying a lower-level discriminator (LLD). The pulse-height spectrum (PHS) is calculated as the average counts of the 64 fibres in each *X* and *Y* plane as a function of LLD, and it is shown in Fig. 8 ▸ by the red and blue curves, respectively. The error bars are calculated as the standard deviation from the mean value for each LLD. They are defined as ±σ and give information about the dynamic range of recorded intensity for each event.

**Figure 9:**
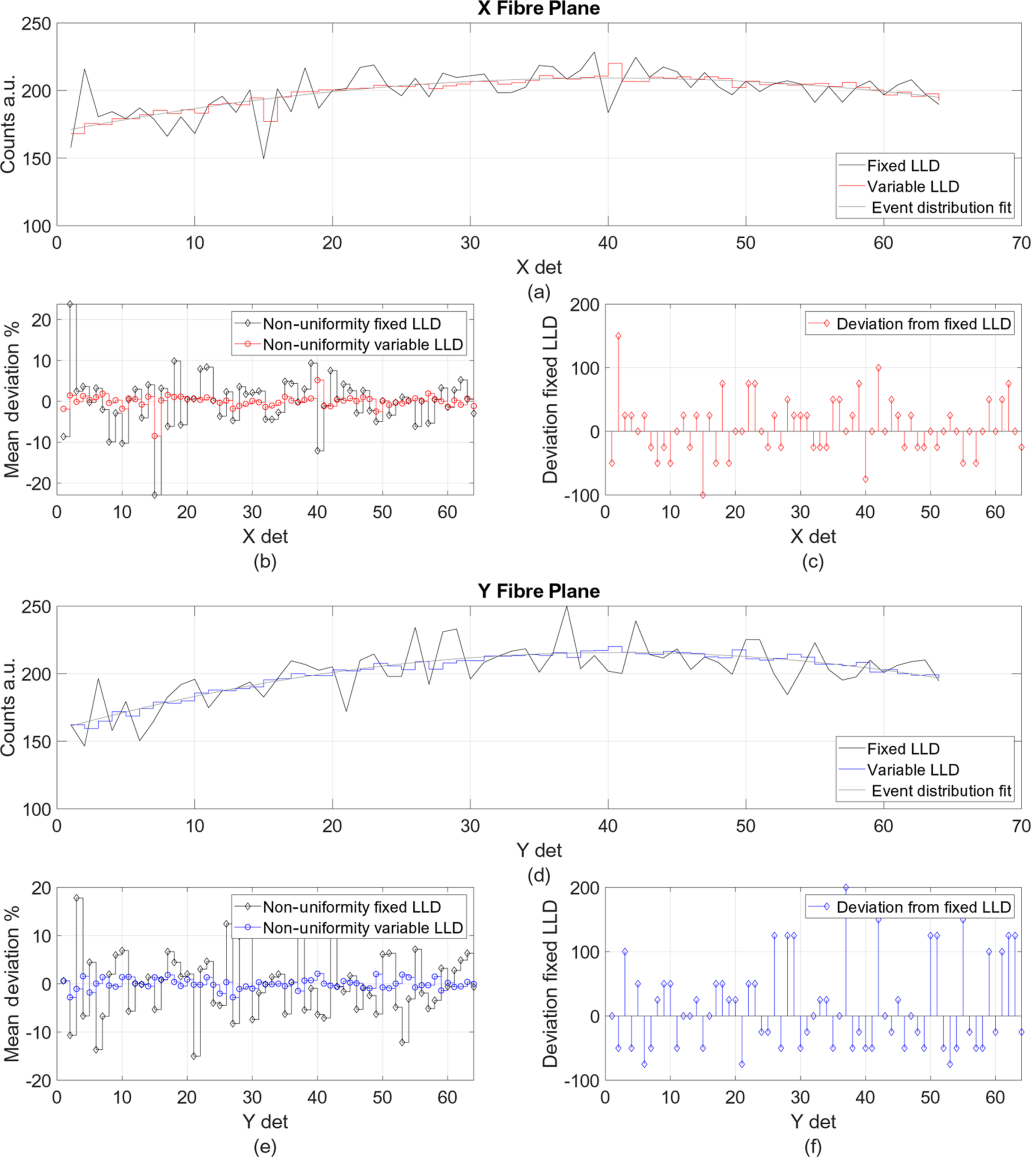
Distribution of counts recorded with a V/Nb sample on the *X* and *Y* fibre planes for fixed LLD (black), variable LLD in (*a*) red and (*d*) blue, respectively, and the calculated event distribution fit in grey. Detector-uniformity variation for each fibre shown as the percentage deviation of the fixed LLD event distribution from the fit in black diamonds and of the variable LLD event distribution from the fit in (*b*) red and (*e*) blue circles for the *X* and *Y* fibre planes, respectively. The uniformity is improved by a factor of 5 when applying the variable LLD correction. (*c*) and (*f*) LLD offset value for each fibre from the fixed LLD = 200, corresponding to offset 0.

It is possible to minimize the non-uniformity of the detector by implementing a PMT channel-to-channel or fibre-to-fibre threshold correction. The one-to-one fibre-to-PMT-channel coding makes the variable-threshold correction straightforward with respect to more complicated fibre-coding coupling methods. The variable-threshold correction procedure was tested by measuring a V/Nb spherical sample on SXD. The 4π scattering from the sample gives a uniform spatial distribution resulting in a parabolic shape across the detector active area. Data were recorded via the FPGA firmware on the basis of single-photon counting for each MAPMT: therefore, for the *X* and *Y* fibre planes independently. The distribution of counts recorded with the fixed LLD at 200 (a.u.) is shown in Figs. 9 ▸(*a*) and 9 ▸(*d*) as the black curve for the *X* and *Y* fibre planes, respectively. A second-order polynomial fit was applied to the data using the least-squares method to obtain the trend of the event distribution. The fit is depicted by the grey line in Figs. 9 ▸(*a*) and 9 ▸(*d*). The deviation from the fit for each fibre is indicated by black diamonds in Figs. 9 ▸(*b*) and 9 ▸(*e*). A detector uniformity variation of ±10% for more than 90% of the detector pixels is measured. This is within current instrument requirements, but there is an opportunity to significantly improve the detector uniformity. The variable LLD correction consists of finding for which LLD value the difference between the calculated fit (*C*_fit_) and the counts recorded per fibre as a function of LLD is the smallest. 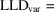 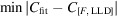 , where *C*_[*F*, LLD]_ represents the data recorded per fibre (*F*) for each threshold value (LLD). The LLD offsets, from the fixed LLD = 200, vary between −100 and 200 and are shown in Figs. 9 ▸(*c*) and 9 ▸(*f*) for the *X* and *Y* fibre planes, respectively. The event distribution based on the variable LLD correction is shown in red in Fig. 9 ▸(*a*) for the *X* fibre plane, and in blue in Fig. 9 ▸(*d*) for the *Y* fibre plane. The detector uniformity is improved by a factor of 5, resulting in a variation of ±2% from the calculated fit. The percentage deviation for each fibre is depicted in Figs. 9 ▸(*b*) (red circles) and 9 ▸(*e*) (blue circles).

## 4. Conclusions

ISIS continues to develop ZnS:Ag/^6^LiF WLSF scintillator detectors to cope with the renewal and upgrade of its suite of neutron-scattering instruments. A novel 2D position-sensitive detector has been developed for single-crystal diffraction applications. The SXD instrument upgrade project foresees the replacement of all 11 clear-fibre detector modules with WLSF modules of the type presented in this work. The detector for this upgrade was produced and tested on ISIS beamlines. The detector design has been described, highlighting the double-layer fibre-scintillator structure for high-efficiency neutron detection. The detector efficiency was investigated both with a prototype detector and with the novel double-layer module and compared with the clear-fibre scintillator detector currently installed on the SXD instrument. With the double-layer detector, more than a factor of 3 and up to a factor of 4 improvement in efficiency was achieved at 2 and 1 Å, respectively. The results obtained from the peak intensity of Bragg reflections of an NaCl crystal on SXD are in agreement with the theoretical calculations and efficiency measurements obtained with the prototype detector. An absolute efficiency of about 80% at 2 Å is achieved with the double-layer detector. The efficiency improvement will enable measurement of smaller samples and characterization of structures of increasing complexity. Further improvements to both position sensitivity and uniformity have been demonstrated. A position-interpolation algorithm (CoG), previously developed for linear WLSF detectors (Mauri *et al.*, 2021 ▸), has been adapted and applied to 2D detectors. Results show a better position resolution with more than a factor of 2 improvement over the standard data acquisition. A detector-uniformity variation of about ±10% was measured with the double-layer WLSF detector. A variable-threshold correction was implemented to minimize the non-uniformity. The correction method has been discussed and a ±2% uniformity variation was achieved. The improvements in both spatial sensitivity and uniformity lead to a better and more-precise peak positioning. A better uniformity response can enhance the discrimination of low-intensity signals from the background. Implementation of both methods in an FPGA for on-board real-time calculation is underway. Several ZnS:Ag/^6^LiF WLSF scintillator detectors have been developed and employed on neutron instruments at ISIS for over a decade, demonstrating an excellent long-term stability and reproducibility (Rhodes *et al.*, 2004 ▸; Sykora *et al.*, 2015*b* ▸). This new high-efficiency double-layer WLSF detector concept enables upgrades to the ISIS instrument suite and extends the scientific capabilities of the facility. 

## Figures and Tables

**Table 1:** Comparison of detector characteristics between the clear-fibre detector currently installed on SXD and single- and double-layer WLSF detectors (with 0.45 mm-thick scintillator sheets)

	Clear-fibre detector	Single-layer WLSF detector	Double-layer WLSF detector
Neutron detection efficiency at 1.8 Å (%)	20	64	84
Local peak rate (kcps)	5	16	16
Gamma sensitivity	<10^−6^	<10^−6^	<10^−6^
Pixel size (mm)	3 × 3	3 × 3†	3 × 3†
		1.5 × 1.5† (with CoG)	1.5 × 1.5† (with CoG)
Active area (mm)	192 × 192	192 × 192	192 × 192
No. of fibres	16384	128	256
No. of PMTs	32	2	2
Approximate weight (kg)	70	6	8
Approximate cost† (GBP)	50000	18000	20000

† At the time of writing.

**Table 2:** FWHM of the Gaussian fits calculated in the *Y* direction for *Y* = 8 and in the *X* direction for *X* = 7, *X* = 9 and *X* = 11, obtained for the raw data and the data analysed with CoG position interpolation The FWHM is expressed as the number of 3 × 3 mm detector pixels. More than a factor of 2 improvement can be achieved by applying the CoG position-interpolation algorithm.

Peak	*Y* = 8	*X* = 7	*X* = 9	*X* = 11
FWHM raw data	1.9	1.7	2.5	
FWHM CoG	0.9	1.3	0.9	1.1
